# A Case of Multi-Organ Failure Secondary to Malabsorption Occurring in the Presence of Acute-on-Chronic Pancreatitis

**DOI:** 10.7759/cureus.48274

**Published:** 2023-11-04

**Authors:** Saira Dar, Kamran Hassan Dar, Umer Hussain, Iqra Rafiq, Hafiz Muhammad Usama Zuhair

**Affiliations:** 1 Family Medicine, Doctors Medical Center, Modesto, USA; 2 Medicine, Faisalabad Medical University, Faisalabad, PAK; 3 Internal Medicine, Faisalabad Medical University, Faisalabad, PAK

**Keywords:** protein energy malnutrition (pem), abnormal weight loss, acute-on-chronic pancreatitis, anorexia nervosa (an), total parenteral nutrition (tpn)

## Abstract

This case report highlights a concerning and complex case of a middle-aged female presenting with severe malabsorption, diarrhea, and subsequent malnutrition. The patient's weight dramatically dropped from 85 lbs to 50 lbs over the course of two to four months. The medical history included ongoing pancreatitis, esophageal ulcers, and previous surgeries for a dermoid cyst in the brain and cervical neoplasia. Upon admission to the hospital, the patient received total parenteral nutrition (TPN) on the first day, but this led to delirium due to refeeding syndrome. Refeeding syndrome is a well-known condition that can occur when malnourished individuals receive too much nutrition too quickly, causing metabolic imbalances and potentially serious complications. Subsequently, during the second hospitalization, the patient did not receive TPN but was instead administered 5% dextrose with 20 mEq of potassium chloride (KCl). Unfortunately, her condition worsened, leading to multiorgan failure. During the third hospitalization, TPN was reintroduced under consultation and hospitalist evaluation, and the patient's symptoms improved.

Overall, this case report outlines a complex case with multiple medical issues, including severe malnutrition, which required careful management and consideration of the patient's unique needs. It underscores the importance of cautious nutritional support for severely malnourished individuals to avoid complications such as refeeding syndrome. The case also emphasizes the value of interdisciplinary collaboration and continuous monitoring to achieve successful outcomes in such complex situations.

## Introduction

Common clinical symptoms, which are a part of various conditions, sometimes overwhelm the clinical picture without substantial evidence to link them to a particular disease or syndrome. Malabsorption and diarrhea are at the core of various diseases and syndromes, with accompanying symptoms such as weight loss, lethargy, and malnutrition.

This report describes the case of a middle-aged female with severe malabsorption, diarrhea, and, in turn, malnutrition who had multiple active diagnoses that complicated the clinical picture as well as posed a diagnostic challenge to the physicians. We come across these symptoms in various forms now and then, and it is important to identify their pathogenesis to treat them effectively. Malabsorption alone can stem from various mechanisms; thus, clear identification is key to initiating appropriate treatment.

Enteropathy is characterized by malabsorption-related symptoms such as (severe) persistent diarrhea, weight loss, and vitamin deficiencies. In extreme situations, parenteral nutrition and immunosuppressive medication are required. The range of immune-mediated enteropathies has grown significantly in recent years, and the current report is also an example of such an unusual enteropathy.

## Case presentation

A female in her late 40s was admitted to the hospital after a ground-level fall due to severe malnutrition that presented with malabsorption, malnutrition, weight loss, and proximal muscle weakness. Along with these, she had severe diarrhea, constituting 10-15 bowel movements per day. Consequently, her normal baseline weight of 85 lbs was reduced to 50 lbs over two to four months, and her BMI was severely reduced to 11 kg/m^2^, requiring assistance to stand and being unable to perform daily activities. She had ongoing pancreatitis with persistently high lipase on labs, which may be due to malnutrition [1]. She also had esophageal ulcers and had a medical history of brain surgery (a dermoid cyst in the temporal lobe performed three times) and cervical neoplasia with human papillomavirus (HPV) treated with cryotherapy.

She underwent an extensive workup, including an MRI of the brain which showed no acute findings, just right temporal macrocystic encephalomalacia after prior dermoid cyst removal, an esophagogastroduodenoscopy (EGD) which showed an esophageal ulcer and she was started on Protonix and Carafate, colonoscopy which was unremarkable with signs of polypectomy), normal cancer antigen (CA) 125 test, mildly elevated carcinoembryonic antigen (CEA), a CT scan of the chest, abdomen, and pelvis which showed no evidence of malignancy, a negative *Clostridium difficile* (*C. difficile*) culture report, an echocardiogram (echo) which showed mild to moderate pericardial effusion, negative celiac work-up, elevated cortisol level of 31 mcg/dL (this was discussed with endocrinology and not thought to have Cushing's disease), and negative antinuclear antibodies (ANA). Her baseline hemoglobin level was 9 g/dL with a normal mean corpuscular volume (MCV); she later developed transaminitis, increased lipase, creatine kinase, and prothrombin time (PT)/partial prothrombin time (PPT) with an international normalized ratio INR of 1.6, probably secondary to persistent malnutrition. Iron was 89 mcg/dL, ferritin was 990 ng/mL, and transferrin saturation was 61% at admission, as reported in Table 1.

**Table 1:** Laboratory values of the different investigations conducted on the patient

Component	Patient value	Reference range
Alanine transaminase (ALT) (serum glutamic pyruvic transaminase (SGPT)), serum/plasma	399 U/L	10 - 35 U/L
Aspartate aminotransferase (AST) (glutamic-oxaloacetic transaminase (SGOT)), serum/plasma	295 U/L	10 - 35 U/L
Lipase	513 U/L	13 - 60 U/L
Prothrombin time	19.7 seconds	12.4 - 14.8 seconds
International normalized ratio (INR)	1.6	0.9 - 1.1
Creatine kinase	658 U/L	>170 U/L
Iron, total	89 ug/dL	37 - 145 ug/dL
Total iron binding capacity (TIBC)	146 ug/ dL	250 - 425 ug/ dL
Transferrin saturation	61%	15-50 %
Ferritin	990 ng/ml	13-150 ng/mL

**Figure 1:**
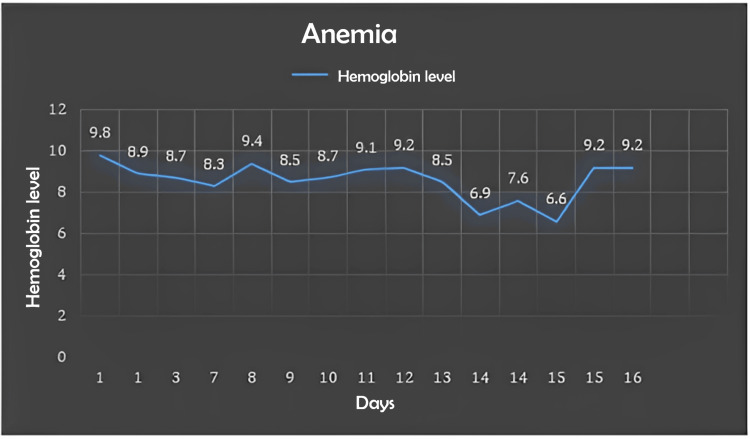
Trend of hemoglobin levels on repeated labs during treatment days

**Figure 2:**
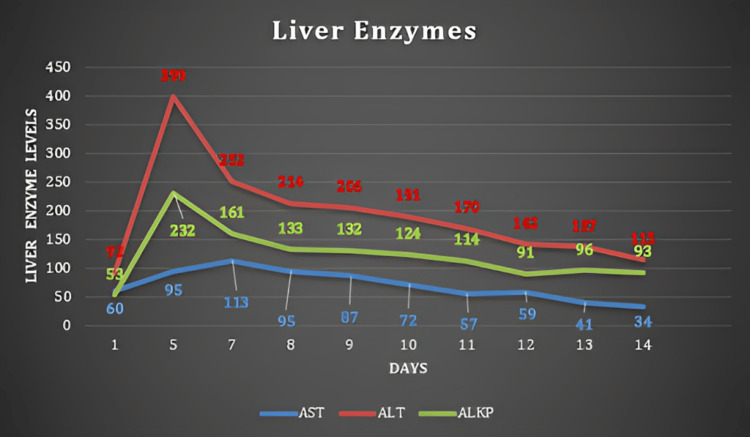
Trend of lever enzymes on repeated labs during treatment days AST: aspartate aminotransferase; ALT: alanine transaminase; ALKP: alkaline phosphatase

Similarly, Figures 1-2 show the wide variations in hemoglobin levels and liver enzymes across investigations.

**Figure 3:**
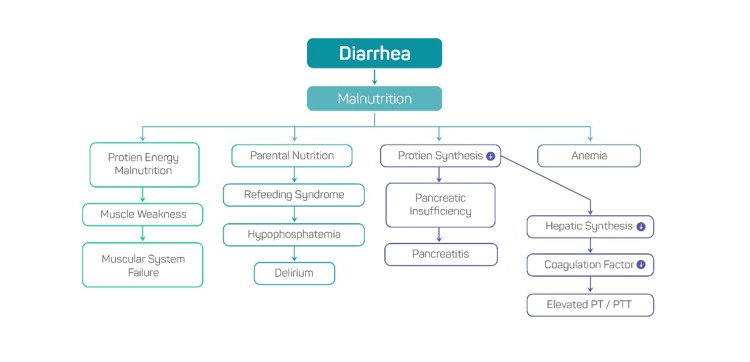
Summary of disease progression Self-produced flow diagram to depict the progression of the disease

The absence of distorted body image per her description and also the absence of fear of gaining weight with total parenteral nutrition (TPN) and welcoming it also did not fully fit anorexia nervosa criteria and it was ruled out on psychiatric assessment. On physical examination, the proximal muscles' strength in the upper and lower extremities was between one and two on a five-point scale, while the distal muscles' strength was between three and five on the same scale, and hand grip and strength were one, with the face swollen. It becomes a further complex and unique case when it progresses to multiorgan failure involving the pancreas, liver, intestine, brain, thyroid, and muscular system in the absence of any remarkable gastrointestinal pathology. Figure 3 gives a summary of the progression of the disease.

During the first hospitalization, the patient was admitted for nutritional support, severe weight loss, and abnormal lab values. Total parenteral nutrition was started. The patient developed delirium on the second day of starting TPN due to refeeding syndrome [2]. She remained delirious approximately 70% of the time while TPN was being given over the next five days. The patient was nevertheless discharged home on day seven, with plans to continue TPN at home. However, it could not be arranged. The patient stayed home for about a week and continued to have episodes of delirium with severe diarrhea and further weight loss before finally getting admitted to another hospital for nutritional support and a workup. Total parenteral nutrition was not given. Instead, 5% dextrose with 20 mEq of potassium chloride (KCl) was given. The patient was encouraged to eat but could not eat much. Multiple labs were done: CBC, comprehensive metabolic profile (CMP), urinalysis (UA), thyroid-stimulating hormone (TSH), lipase, amylase, and creatine kinase. An MRI of the abdomen showed ascites, a dilated common bile duct (CBD), the head of the pancreas that could not be seen, a loss of fat pad at the superior mesenteric artery (SMA), and an echocardiogram showing small to moderate pericardial effusion. The electroencephalogram (EEG) showed foci of activity but was not consistent with epileptiform activity. Based upon consultations and hospitalist evaluation, it was decided that TPN should be restarted at home, and the rest of the management could be done on an outpatient basis. The patient was discharged after one week. At home, TPN could not be started, and the patient's condition worsened. The patient was admitted to another hospital for the third time for ongoing weakness and weight loss. Total parenteral nutrition was started. Labs and previous hospital records were reviewed. There was an improvement in symptoms of delirium after TPN was started. Labs were thoroughly monitored, and the TPN formula was modified based on lab results. Her blood reports were as follows: CBC: hemoglobin (Hb) dropped to 6.6 g/dL but then improved to 9.2 g/dL after one unit of packed RBCs; CMP: electrolytes improved after correction in the TPN formula, PT, and INR; and other labs improved slightly (Table 2).

**Table 2:** Investigation results show improvement in labs after treatment.

Improved labs		
Component	Value	Reference range
Alanine transaminase (ALT) (serum glutamic pyruvic transaminase (SGPT)), serum/plasma	142 U/L	10 - 35 U/L
Aspartate aminotransferase (AST) (glutamic-oxaloacetic transaminase (SGOT)), serum/plasma	59 U/L	10 - 35 U/L
Prothrombin time	16.1	12.4 - 14.8 seconds
International normalized ratio (INR)	1.2	0.9 - 1.1
Hemoglobin	9.4 mg/dl	11.7 - 15.7 g/ dL

The electrocardiogram (EKG) was normal. The patient continued to have muscle weakness and was discharged home after one week with arrangements to continue TPN at home. The patient resumed TPN at home along with lab monitoring. Physical and occupational therapy was started, and the condition of the patient improved.

## Discussion

This case suggests that the range of enteropathies has grown. Because these enteropathies share several histological and clinical characteristics, distinguishing them might be difficult [3]. The majority of the invasive and non-invasive tests, including tissue biopsies from gastrointestinal mucosa and radiological investigations, reveal non-specific changes, thus making a diagnosis not easy. Due to the diagnostic difficulties and rarity of the condition, very few similar cases of malnutrition associated with chronic diarrhea are reported. One is reported in China, where a 44-year-old male complained of chronic diarrhea. Over the previous five years, the patient had intermittent attacks of watery diarrhea, tiredness, and 15-kg weight loss [4]. Similarly, in Germany, three cases of severe Olmesartan-related chronic diarrhea accompanied by weight loss and malassimilation syndrome were reported. Histologically, each patient had a sprue-like enteropathy, and although serological tests for celiac disease were negative [5], none of these cases progressed to multi-organ failure.

Initially, this case was treated as a case of severe protein malnutrition secondary to anorexia nervosa, and TPN was started. Later on, during a psychiatric evaluation, it was discovered that there was an absence of anxiety about gaining weight with TPN, and its acceptance, which ruled out anorexia nervosa [6].

The gut immune system must be able to distinguish between harmless and hazardous antigens since ongoing exposure to the environment can either cause tolerance or the development of an immune response [7]. Immune-mediated enteropathies can result from overreacting intestinal immune cells to non-pathogenic substances (IME). A response like this could result in the breakdown of the mucosal surface, which would then produce the clinical symptoms of a malabsorption condition. For a long time, celiac disease has been acknowledged as the most prevalent IME in adults [3]. The patient underwent a workup for chronic diarrhea, with labs overall unremarkable with a prior EGD/colonoscopy and a biopsy negative for inflammatory bowel disease (IBD) or other pathology and a negative ANA rule out these autoimmune diseases.

We ruled out all potential causes of malnutrition in our patient, including celiac disease, lymphoma, ulcerative colitis, and Crohn's disease. Colonoscopic biopsies found no signs of inflammatory bowel disease, bowel cancer, or malabsorptive syndromes. The absence of parasites and fat globules in the feces allowed for the conclusive exclusion of steatorrhea and parasitic diseases, respectively. The results of the small intestinal fluid culture ruled out small intestinal bacterial overgrowth. Retroviral studies were conducted, but HIV was negative.

## Conclusions

In this case report, a middle-aged female presented with the chief complaints of ground-level falls due to malnutrition, severe weight loss, and proximal muscle weakness. The clinical condition points towards anorexia, celiac disease, or immune-mediated enteropathies; all of these were ruled out in investigations. This case is rare, as there are conditions where malnutrition affects one or two organs directly associated with it but never progresses to multi-organ failure. This case is unique because very few cases of such presentations have been reported. Through this case report, we intended to highlight the range of enteropathies that continues to increase, and also that the management of such complex and complicated cases requires treatment to start with small goals, achieve them, and optimize symptoms. Such scenarios often require extensive monitoring; a carefully planned and informed approach would benefit both the patient and ease the healthcare burden as well.
